# Fine-scale spatiotemporal influences of salmon on growth and nitrogen signatures of Sitka spruce tree rings

**DOI:** 10.1186/1472-6785-13-38

**Published:** 2013-10-04

**Authors:** Thomas Edward Reimchen, Caroline Hazel Fox

**Affiliations:** 1 Department of Biology, University of Victoria, Station CSC, Victoria, PO Box 3020 V8W 3N5, BC, Canada; 2 Raincoast Conservation Foundation, Sidney, P.O. Box 2429 V8L 3Y3, BC, Canada

**Keywords:** Spatial subsidy, Salmon, Nitrogen, Sitka spruce, Conifer, Riparian, Stable isotope

## Abstract

**Background:**

The marine-terrestrial transfer of salmon (*Oncorhynchus spp.*) provides a substantial pulse of nutrients to receiving ecosystems along the Pacific coast of North America and has been shown to enhance productivity and isotopic signatures of conifers and other riparian vegetation. An explicitly spatial, within-watershed investigation of the influence of salmon on conifers has never been previously investigated. In a small salmon-bearing watershed in Haida Gwaii, Canada, the transfer and distributional pattern of salmon carcasses into the riparian zone by black bears provided a spatial basis for investigating the influence of salmon on Sitka spruce tree ring growth and nitrogen isotopic signatures (δ^15^N) across a gradient of salmon carcass densities in relation to salmon escapement.

**Results:**

Annual growth was found to be highest in the high salmon carcass zone and δ^15^N signatures closely tracked the known distribution of salmon carcasses at distances into the forest and upstream. Tree diameter demonstrated a positive relationship with δ^15^N signatures for trees with and without salmon carcass influence. Using an information theoretics approach with general linear mixed models (GLMMs), we show that salmon abundance, mean annual temperature and the interaction terms salmon abundance*temperature and salmon abundance*distance into the forest best predict tree growth. In addition, spatial variables (distance into forest and upstream) and their interaction are the strongest predictors of δ^15^N signatures. However patterns observed in individual trees, particularly those at increased distance into the forest, suggest positive relationships with historical salmon abundance.

**Conclusions:**

Using a replicated spatial sampling design across a sharp gradient in salmon nutrient loading, our study provides clear evidence that the temporal pattern in an allochthonous nutrient source and an interaction with temperature and spatial location influences conifer growth. Although salmon abundance has been previously linked to annual conifer growth and δ^15^N levels, our approach demonstrates the need to incorporate additional predictors including tree size and opens up the prospect of their dual use as historical proxies for salmon abundance.

## Background

Ecological linkages between marine and terrestrial communities are important processes structuring coastal ecosystems. One of the most geographically broad linkages that has received increased attention in recent decades is the role of migrating salmon (*Oncorhynchus spp.)* to coastal watersheds [1]. In addition to the contribution of salmon carcasses to primary productivity in lakes and streams [2,3], salmon also comprise a substantial component of the diet in a diversity of marine and terrestrial taxa including pinnipeds and bears as well as an extensive history of use by First Nations [4]. Despite the increased attention and numerous publications, there remains ambiguity and large knowledge gaps regarding the relationship between salmon and receiving ecosystems.

During an investigation from 1992 to 1994 at Bag Harbour, an intact salmon watershed on the southern reaches of Haida Gwaii, western Canada, it became evident that foraging black bears (*Ursus americanus*) transfer large numbers of salmon from the stream into the riparian zone and uneaten remnants of the carcasses accumulate on the forest floor throughout the spawning period [5]. These carcass remnants were widely distributed but the majority occurred within 50 m of the stream with densities averaging 0.2 carcasses/m^2^ adjacent to the spawning zones. Multiple scavengers including flocks of corvids and dipteran larvae utilized and further dispersed these nutrients [6]. Subsequent investigations in other watersheds of coastal British Columbia confirmed widespread transfer of salmon nutrients to the riparian zone by bears [7] as well as a utilization of these nutrients by riparian vegetation [8-10], insects [11,12] and songbirds [13]. Studies in Alaskan watersheds have identified both flooding and hyporheic movement as well as predator activity as a source for uploading of salmon nutrients [14-16].

The ecological consequences of salmon-derived nutrients to riparian vegetation include increased plant growth rates [17,18], enrichment of foliar nitrogen [8,10,14] and altered plant community diversity [8,10,19]. Because nitrogen is often a limiting nutrient in temperate forests of the Pacific Northwest [20], it is possible that short and/or long term differences in salmon returns to spawning rivers would co-vary with primary productivity in the riparian zone and if so, annual growth rings of these old-growth trees might contain historical information on relative abundance of salmon. The recent identification of nitrogen isotopes in small quantities of wood from individual tree rings [9] offers an additional direct assessment of the isotopic enrichment of marine-derived nutrients over time [21].

We extend the investigation at Bag Harbour and examine the potential influences of salmon nutrients on yearly growth in Sitka Spruce, a dominant riparian conifer, through analyses of tree rings. Recent studies suggest that there is limited potential of tree rings for interpreting historical trends in salmon abundance due to the complexity of edaphic and climatic processes influencing tree growth, the extent of nitrogen uptake and translocation among rings as well as stable isotope fractionation [18,22-25]. However, the distribution of carcasses has not been quantified in previous studies of riparian growth nor has a fine-scale (within watershed) examination of both tree growth and δ^15^N in relation to salmon been previously undertaken. Recently, Drake and Naiman [18] provide comparisons across multiple watersheds yet control sites were for 2 to 25 km distance from salmon sites with no exploration of spatial or temporal within-stand relationships to salmon abundance. We established a sampling grid adjacent to Bag Harbour stream between the estuary and the headwater lake that encompassed the previously identified distribution of salmon carcasses [5,6]. Thirty-six of the largest Sitka Spruce (*Picea sitchensis*) located over a range of carcass densities throughout this grid were cored and subsequently quantified for yearly radial growth. Yearly abundance of salmon in this watershed has been recorded (Department of Fisheries and Oceans Canada) from 1947 through to the present and varies by more than an order of magnitude among years (range 400-35,000) and consequently, our results and analyses are restricted to this time period.

In this study, our objectives are to: 1) characterize the spatial pattern of annual growth (tree ring width) and nitrogen isotopic signatures (δ^15^N) in trees across replicated gradients of salmon carcass densities, 2) identify the temporal signatures (1947-2000) of radial growth and nitrogen isotope signatures in relation to known yearly salmon escapement and 3) assess the predictors of both tree ring growth (index) and δ^15^N levels using an information theoretics approach that relies on general linear mixed models (GLMMs). Potential predictors of growth and δ^15^N values in annual tree rings included environmental (temperature, precipitation and salmon abundance), spatial (distance into forest and distance upstream) and total nitrogen as variables. Our replicated transects perpendicular to the stream channel across sharp gradients in carcasses and throughout the length of the stream along a broad range of salmon spawning densities allowed multiple paired comparisons of growth and nitrogen isotope signatures of trees. Although not directly exploring the use of tree ring data as a proxy for historical salmon abundance in this study, our results are useful for evaluating tree ring growth and δ^15^N values as potential proxies.

## Methods

### Study area

**Figure 1:**
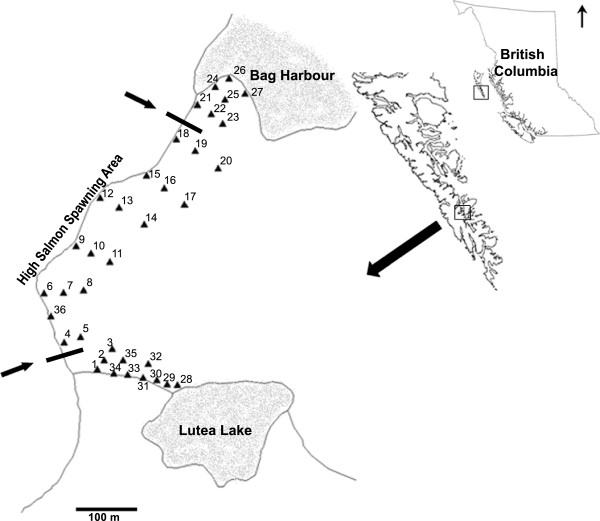
**Study area, located on Haida Gwaii, British Columbia, Canada, 36 tree core locations (closed triangles) and high salmon spawning area.** Numbers refer to tree core identities.

Bag Harbour watershed (52˚20′40 N, 131˚22′15 W), located in southeastern Haida Gwaii, British Columbia, Canada (Figure 1), is largely composed of old-growth forest, mainly Sitka spruce (*Picea sitchensis*), Western hemlock (*Tsuga heterophylla*) and western redcedar (*Thuja plicata*). Alder (*Alnus* spp) are uncommon in the riparian zone of this watershed. One salmon-bearing stream, which ranges from 5 to 20 m in width and usually less than 0.5 m depth, is fed by a small lake, located ~1.2 km upstream from the estuary. The majority of salmon spawn in gravel beds located between 100 m and 800 m upstream, with limited spawning occurring above 800 m. Chum (*Oncorhynchus keta*) make up the majority (>90%) of spawning salmon, although smaller numbers of coho (*O. gorbuscha*) and pink (*O. kisutch*) salmon are also present (data from Fisheries and Oceans Canada). Black bears (*Ursus americanus*) are the dominant vector of salmon from the stream to the forest floor and a diversity of species utilize the abandoned carcasses [6].

### Field methods

**Figure 2:**
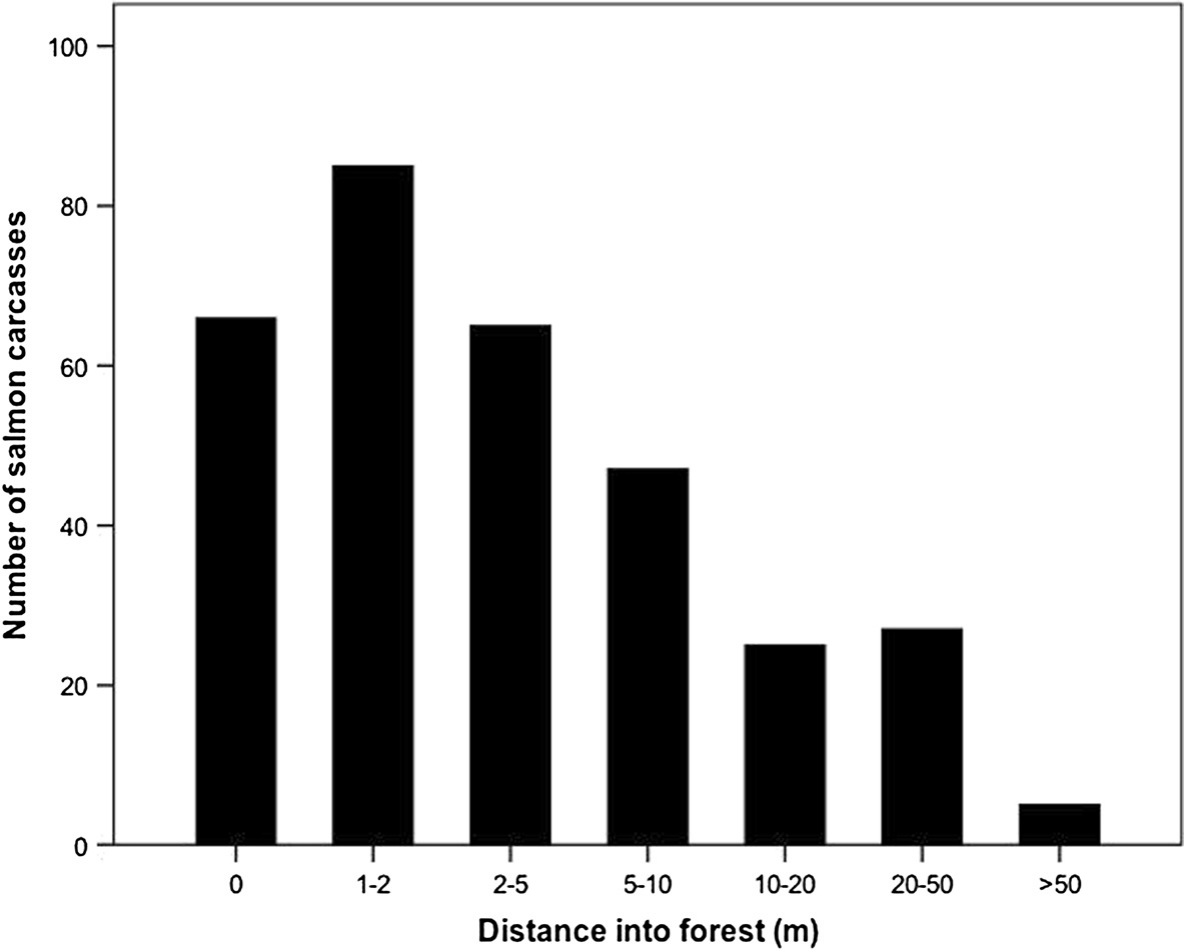
**Average distribution of bear-transferred salmon carcasses with distance (m) into the forest at major spawning reach at Bag Harbour, Haida Gwaii.** Information was obtained from searches of the forest areas adjacent to spawning gravels, below 800 m upstream (data from Reimchen 1994).

In fall 2000, a riparian sampling grid was established across a previously documented gradient of high to zero salmon carcass densities and extending in a 100 m band along the stream from the estuary to the lake [5,6] (Figure 2). At each of 15 sites along the length of the stream, separated by approximately 100 m, the largest Sitka spruce closest to the stream (usually within 10 m) was chosen for coring. At 11 of these sites, one or two additional of the largest spruce were cored along a perpendicular transect into the forest, the first near the outer edge of the distribution of salmon carcasses (~50 m) and the second, when present, approximately twice the distance from the stream and typically comprising the most distant spruce identified from the stream. Spruce were less abundant above the spawning reaches of the stream and when present were close to the stream channel. We used the presence or absence of salmon carcasses as a general proxy for the spatial input of salmon nutrients into the forest. We refer to trees located at sites without evidence of carcasses (i.e. those above the spawning reaches and those located most distant into the forest) as ’control or reference’ trees. These trees reflect low to trace salmon-nutrient loading.

### Tree core sampling and growth index

Each spruce was cored at breast height using a 1.2 cm × 40 cm increment borer, measured for diameter at breast height (dbh) and distance from the stream and distance upstream from the estuary. Presence or absence of salmon carcass remnants around the base of the tree (~ 5 m radius) was recorded. Cores were oven dried at 60^o^C for at least four weeks, mounted on plastic sheaths, sanded with fine-grade sandpaper and digitally scanned. Images of cores were used to individually measure tree ring widths to the nearest 0.001 mm using Coorecorder (http://www.cybis.se) with problematic rings visually assessed under a dissecting microscope. Tree ring series were crosschecked for accuracy using default settings in COFECHA [26]. Individual tree ring series were then fitted to a single smoothing spline with a 50% frequency cutoff of 100 years, a procedure that removes age-related growth trends in individual series, using the program ARSTAN [27]. The use of a 100-year spline as opposed to the default 32-year spline is that mid to high frequency variations are retained in trends less than 100 years and long-term growth related trends are removed (e.g. [18]). We refer to this metric as the ARSTAN-modified growth index or growth index in subsequent analyses.

### Stable isotopes

Stable isotope samples were collected by excising individual tree rings from dried cores using a scalpel blade. When rings were very small (<0.5 mm) and there was insufficient mass for analyses, samples from two or more sequential years were combined. We analyzed a relatively complete ring series (1947-2000) on 14 of the trees comprising six paired comparisons (carcass vs control) at different positions (Figure 1) along the length of the stream channel (#18&20, 15&17, 12&13, 9&11, 6&8, 4&5) and two additional control trees (30&28) at 500 and 700 m above the upper reaches of the spawning zones. In addition, we analysed 10 rings from each of 11 additional trees distributed from the estuary to the headwater lake (#24,25,23,19,16,36,2,31,34,29) comprising two time periods (1950-1954, 1975-1979). These two periods were chosen as part of a coastwide assessment of nitrogen isotope signatures in conifer tree rings and characterize the earliest period (50-54) where salmon abundance is known for most watersheds and a recent period (75-79) that largely excludes use of more recent sapwood rings. Tree ring samples were ground for five minutes using a Wig-L-Bug Amalgamator. Samples (30 mg) were packaged in tin capsules and analyzed for total nitrogen and isotopes of nitrogen at the University of California Davis Stable Isotope Facility using a PDZ Europa ANCA-GSL elemental analyzer interfaced to a PDZ Europa 20-20 isotope ratio mass spectrophotometer (Sercon Ltd., Cheshire, UK). Nitrogen isotope results are reported in δ notation (δ^15^N) relative to the international standard of atmospheric N_2_, in parts per thousand. Isotopic abundances are calculated by:

(1) $$\delta^{{}^{15}}N=R_{\text{sample}}/R_{\text{standard}}$$

where R equals the ratio of ^15^ N/^14^ N stable isotopes. Reproducibility of δ^15^N values on replicated wood samples averaged +/- 1.0‰.

### Environmental data

Temporal data included salmon abundance (escapement) data for Bag Harbour (1947-2000; Fisheries and Oceans Canada) modified using a three-year running average from the previous three years. This three-year average with a one-year lag [28] was used as a conservative temporal estimate of potential salmon nutrient input. Precipitation and temperature data were obtained from nearby Sandspit for the majority of years, with data from Tlell or alternatively Masset used when Sandspit data were not available (Environment Canada National Climate Data and Information Archive, http://www.climate.weatheroffice.gc.ca). Total precipitation and mean maximum monthly temperature data were generated from September in the previous growth year to October of the growth year for any given tree ring, as these periods more closely reflect the biological year [29,30].

### Generalized linear mixed models

We assessed the influence of salmon abundance and other potential predictors on tree growth using the ARSTAN-modified growth index and δ^15^N isotopic enrichment separately, using an information theoretics approach. Predictors were selected due to their availability but most importantly, their possible influence on tree growth and δ^15^N values. For both model sets, response variables were first examined with all potential predictor variables to determine the nature of the potential relationship. Predictor variables were also examined for collinearity. Statistical analyses were performed using SPSS v.20 (IBM, USA).

Predictors of the growth index were assessed using General Linear Mixed Models (GLMMs) for the period 1948-2000. Individual trees (n = 36) were specified as random factor subjects with year as the repeated measure. Covariance was specified as AR1 in order to account for the autoregressive nature of the data. Growth index, the response variable, was transformed closer to normality using a Box-Cox transformation and salmon abundance, precipitation and temperature were log transformed. Thirty-two biologically feasible models were constructed using the predictor variables salmon abundance, precipitation, temperature and the interaction with distance upstream (upstream) and distance into the forest (forest). Spatial predictors were only included as interaction terms due to the consequence of ARSTAN growth index, which standardizes tree series, thus removing the main spatial effects of growth, along with age-related trends. The null model (intercept only) was also included. Models were ranked using Aikaike’s Information Criteria for finite sample sizes (AcxxICc). Where more than one model was ranked with an AIC value of >2, model averaging of the confidence set of candidate models was performed on parameter estimates and variances. The confidence set of candidate models was based on those with a cumulative sum of >95% AICc weight.

Predictors of δ^15^N isotopic enrichment for individual tree rings were also assessed using multiple General Linear Mixed Models (GLMMs) for the period 1948-1989 (n = 25 tree cores, n = 681 rings). Tree ring isotopic signatures obtained in years more recent than 1989 were excluded due to elevated total nitrogen and variable δ^15^N isotopic values in the biologically active sapwood (Reimchen and Fox, unpublished data). Individual trees were specified as random factor subjects with year as the repeated measure. Covariance was specified as autoregressive (AR1) in order to account for the autoregressive nature of the data. Distance into the forest and tree dbh demonstrated an unacceptably high level of collinearity. As there was a strong relationship between δ^15^N and tree dbh, we adjusted δ^15^N isotopic enrichment values based on a dbh correction function generated using a linear regression of dbh and the δ^15^N values of all trees:

$${\text{logY}}^{\prime }{}_{\mathrm{ij}}=\left[{\text{log}\;Y}_{\text{ij}}-\left(v_{j}\left(\text{log}\;x_{i}-\text{log}\;x\right)\right)\right]$$

where Y’_ij_ is the adjusted value of δ^15^N for year i, Y_ij_ is the original δ^15^N value, v_j_ is the regression slope, *x*_i_ is the annual radial growth of year i and *x* the δ^15^N signature for the year 2000. We present data for both unadjusted and dbh-adjusted isotopic signatures but only use the latter for analysis using GLMMs.

Dbh-adjusted δ^15^N isotopic signature, the response variable, was transformed closer to normality using a Box-Cox transformation, growth index was log transformed and distance upstream (upstream) was converted to a categorical value (above and below 800 m) due to a non-linear relationship with the response variable. Twenty-three biologically feasible models were constructed using the predictor variables salmon abundance (1 year lag), growth index, percent nitrogen, categorical distance upstream (upstream), distance into the forest (forest) and interaction terms. Interaction terms involving spatial predictors and the growth index were not included. The null model (intercept only) was also included. Models were ranked using Aikaike’s Information Criteria for finite sample sizes (AICc).

## Results

### Spatial trends

**Figure 3:**
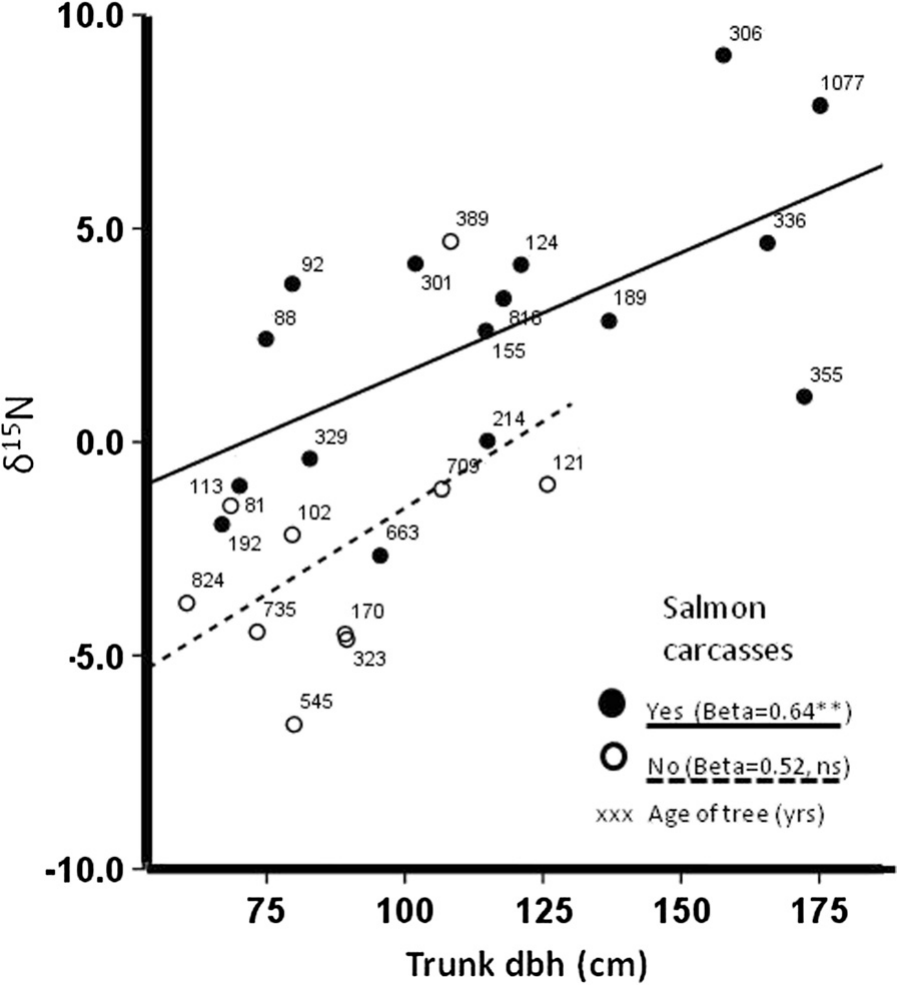
**Relationship between mean tree δ**^**15**^**N values (1975-1980) and trunk dbh (cm) at breast height at Bag Harbour, Haida Gwaii.** Circles represent individuals trees with salmon carcasses present (closed circle) and absent (open circle). Numbers above symbols represent tree age, as determined by coring. Standardized regression coefficients shown for salmon carcass trees and control trees. Statistical probabilities (P < 0.01 & NS) shown for two regression slopes.

In the 15 transects between estuary and the headwater lake, trees in the carcass zone were larger (mean = 393 cm, n = 17) than adjacent control trees (mean = 278 cm, n = 17, t = 3.5, P < 0.001) but did not differ in average age (mean = 380 and 445 yrs respectively, t = 0.6, P = 0.51). The largest average annual growths (all years) were found in trees adjacent to the major spawning reaches of the stream but average growth did not vary with respect to distance into the forest (P = 0.65). Among the 25 trees with stable isotope data, δ15N signatures (1975-1979) ranged from -6.6 to 9.2 among trees, with the highest values occurring with carcasses (mean δ15N = 2.5, n = 15) than in the control trees (mean δ15N = -2.4, n = 10). Data were similar for 1950-1954 (overall range: minus 6.1 to 9.4; carcass trees mean δ15N = 1.0, n = 15, control trees mean δ15N = -4.1, n = 10). We examined the interaction between tree size (dbh) and isotope values and found a significant positive slope (Beta = 0.72, P < 0.001) with a similar trend for both carcass and non-carcass trees although only significant in the former (Figure 3). Percent nitrogen was marginally but non-significantly higher for carcass trees (6.2%) than control trees (5.2%) (F = 1.0, P = 0.31).

### Temporal trends

**Figure 4:**
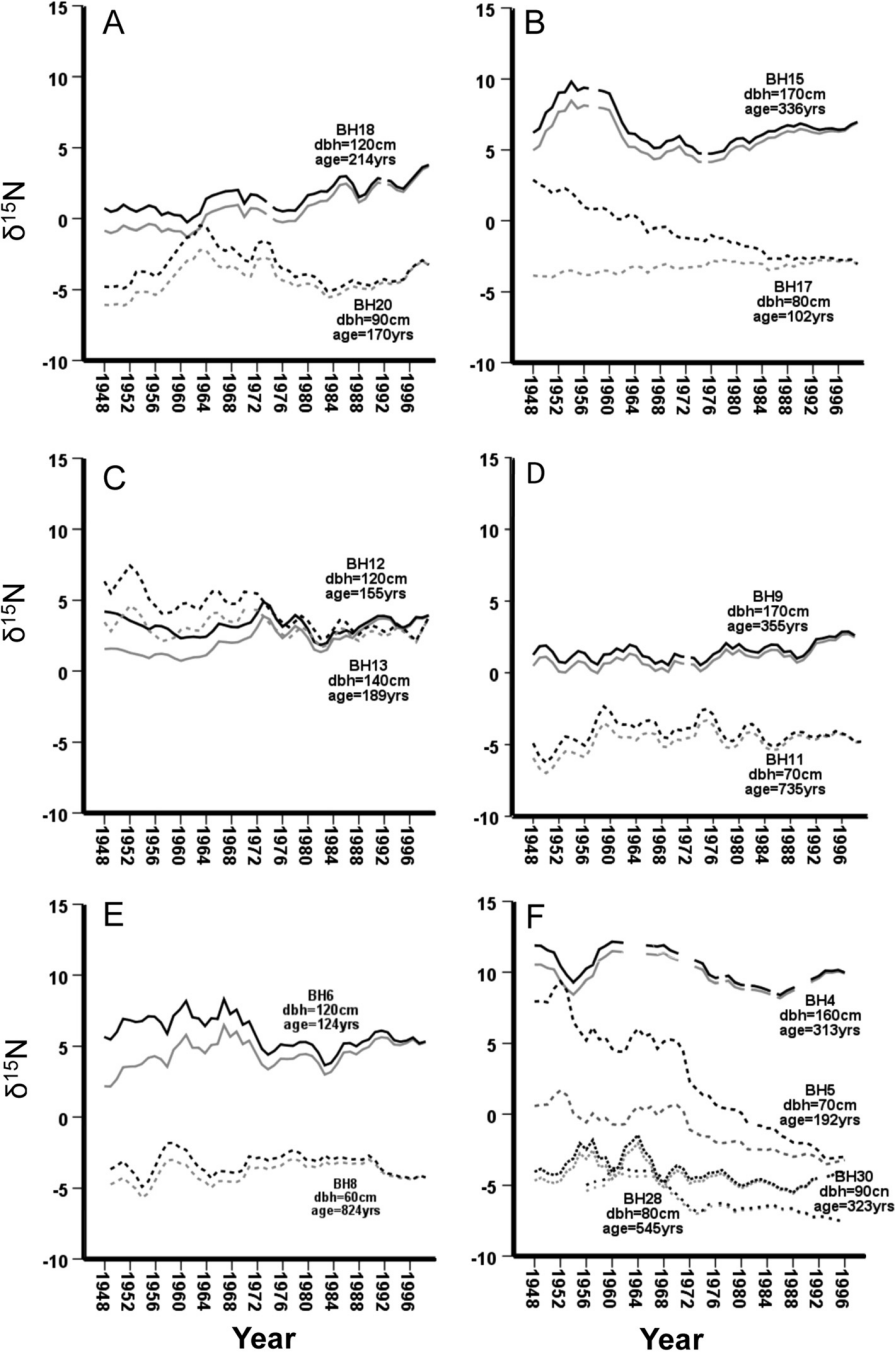
**δ**^**15**^**N isotopic enrichment values (unadjusted-grey line) and dbh-adjusted-dark line) for Bag Harbour trees cored across gradient in salmon carcass density.** Panels organized (top left, top right etc.) for increased distance from the estuary. Panel **A**: BH18 (200 m upstream, 1 m into forest, carcass present) BH20 (200 m upstream, 80 m into forest, carcass absent); Panel **B**: BH15 (300 m upstream, 4 m into forest, carcass present) BH17 (300 m upstream, 90 m into forest, carcass absent); Panel **C**: BH12 (400 m upstream, 1 m into forest, carcass present) BH13 (400 m upstream, 27 m into forest, carcass present); Panel **D**; BH9 (500 m upstream, 3 m into forest, carcass present) BH11 (500 m upstream, 66 m into forest, carcass absent); Panel **E**: BH6 (600 m upstream, 4 m into forest, carcass present) BH8 (600 m upstream, 64 m into forest, carcass absent); Panel **F**: BH4 (700 m upstream, 1 m into forest, carcass present) BH5 (700 m upstream, 48 m into forest, carcass present); BH30 (1000 m, 3 m into forest, carcass absent), BH28 (1200 m upstream, 31 m into forest, carcass absent).

We have relatively complete isotope data (1947-2000) for 14 trees. Within most of these (Figure 4), the unadjusted δ^15^N signatures varied approximately 5‰ over the 53 years with some trees (Figure 4A, BH18, Figure 4D, BH9) showing a depletion historically and others (Figure 4E, BH6, Figure 4F, BH4, BH5, BH30) exhibiting higher signatures in the past. Five of the six trees found further from the stream had lower signatures (5-13‰) relative to trees located closer to the stream, with a single exception (Figure 4C, BH13), but in this case, salmon carcasses were present in both sites. The highest signatures of any tree (BH4, Figure 4F), reaching 12‰, occurred in the major bear-foraging area. These signatures were ~10‰ higher than the corresponding control tree (BH5) 50 m into the riparian zone but this difference was greatly reduced historically as demonstrated by the increased isotopic signatures in the control tree during earlier periods. When we corrected isotope data for size of tree in each year, the temporal trends suggest the control tree increased by 10‰ achieving similar isotopic signatures to BH4 during the earliest sampling period in 1947. Smaller increases (~ 5‰) of size-adjusted values occurred historically in three additional carcass trees (BH15, BH12, BH6) and in three control trees (BH20, BH17, BH28). The lowest unadjusted and adjusted signatures occurred in the two control trees (BH30, BH28; Figure 4F) above the upper extent of the spawning zone and both of these showed a 3-5‰ increase back to 1947. We evaluated the relationship with salmon abundance which fluctuated from ~3000 in the late 1990s to ~30,000 in the late 1940′s. Five of the 14 trees showed a non-significant negative association while nine showed a positive relationship between size-adjusted δ15N and salmon abundance of which four (BH17, BH13, BH5, BH28) were statistically significant (P < 0.05).

### Temporal trends for GLMM variables

**Figure 5:**
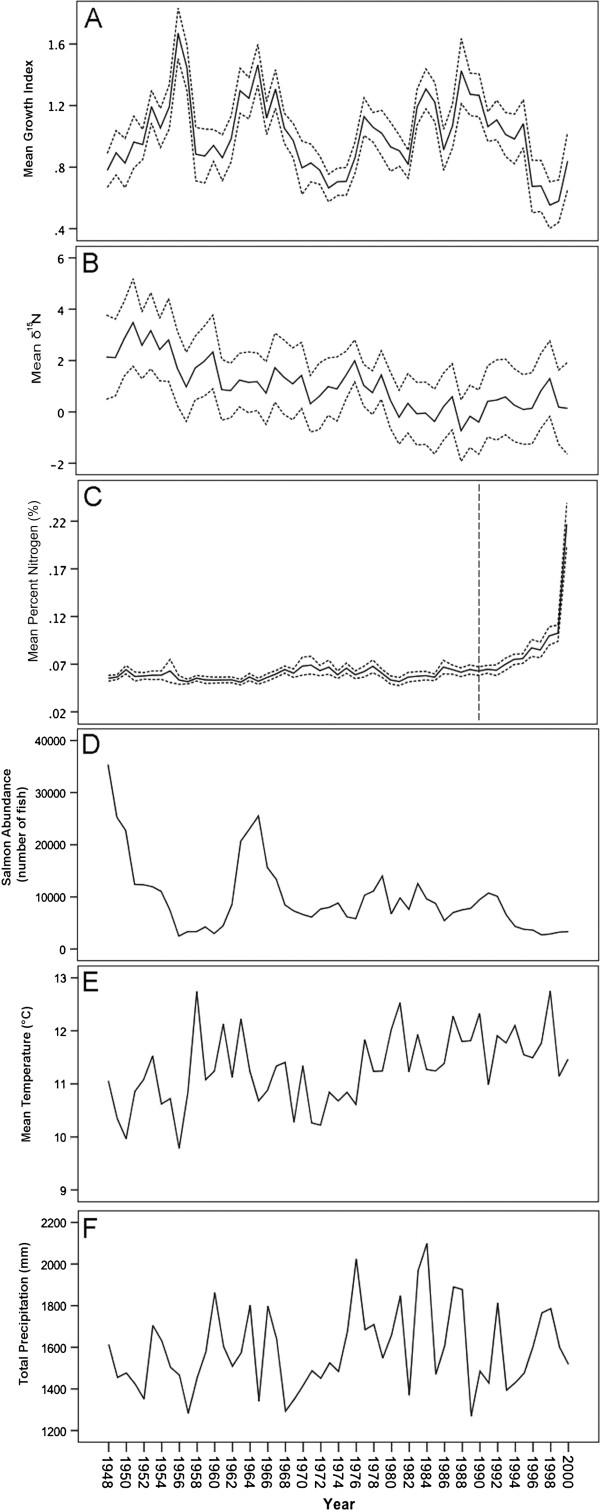
**Yearly trends in radial tree ring growth, nitrogen signatures, salmon abundance, atmospheric temperature and precipitation at Bag Harbour, Haida Gwaii. (**Panel **A)** Mean annual tree growth index, averaged for 36 tree ring series with dashed lines indicating standard error **(**Panel **B)** mean annual δ15N isotopic values for a subset of tree rings (dbh-adjusted) with dashed lines indicating standard error, **(**Panel **C)** mean annual percent nitrogen (%) with dashed lines representing standard error, **(**Panel **D)** annual salmon abundance (3 year running average, lagged 1 year) at Bag Harbour, Haida Gwaii, **(**Panel **E)** mean maximum monthly temperature (°C) and **(F)** total precipitation (mm) from local weather stations (Sandspit, Masset and Tlell, Haida Gwaii) for the biological year (1948-2000). The vertical dashed line in 5C indicates data that was not included in a second δ15N GLMM run using a subset of temporal data (1948-1989).

To provide a visual context for variables used in the GLMMs, we characterize the mean values for standardized growth index, δ15N, percent nitrogen, salmon abundance, temperature and total precipitation (Figure 5). Standardized growth was highly variable, with clear peaks in mean growth over the study period (Figure 5A). Adjusted δ15N was also highly variable with decline in mean values towards the present (Figure 5B). Percent nitrogen showed low variance among years apart from the recent decades which showed a striking increase in percentages concordant with the outer active sapwood (Figure 5C). Salmon escapement fluctuates over time with two large pulses occurring in the late 1940′s and early 1960′s (Figure 5D) and a general decline with time from the 1960′s to the recent. Abundances in Bag Harbour prior to the mid-1940′s are not currently known but are anticipated to have been higher [31]. The environmental variables, mean temperature and total precipitation, were calculated for the biological year. On average, temperatures generally increase with time (Figure 5E) and Bag Harbour and the surrounding area receives between 1200 and 2100 mm of precipitation (mainly in the form of rain) for the biological year (Figure 5F).

### Predictors of growth using GLMMs

Due to a lack of discrimination for the highest ranked model using AICc, the top two ranked models that best predicted the growth index were averaged and the reported cumulative weight (ω_i_ = 0.998) generated using both model terms (Table 1). Leading models include the temporally dynamic predictor variable salmon abundance, temperature and the interaction terms salmon*temperature and salmon* distance into the forest. Summing parameter weights across the candidate set of best-supported models demonstrated the high relative importance of salmon, temperature and the interaction between salmon and temperature; spatial interaction terms with salmon were lesser contributors to the top model set (Table 2). Although parameters estimates were small, particularly for the influence of temperature (Table 3), we note that the growth index is a heavily modified metric and both means and variances are standardized between tree series. Salmon, the interaction between salmon abundance and temperature had positive parameter estimates, as did the interaction between salmon abundance and the distance into the forest. The interactions between [1] salmon abundance and temperature and [2] salmon abundance and distance into the forest were similar, with a larger influence of salmon abundance on growth at higher temperatures and similarly, a larger effect of salmon abundance with increasing distance into the forest (Table 3).

**Table 1:** **Top ten most highly ranked models of indexed annual Sitka spruce growth and δ**^**15**^**N isotopic enrichment**

**Response variable**	**Model rank**	**Model predictors**	**AICc**	**Δ AICc**	**ω**_**i**_
Growth index					
	1	salmon, temperature, salmon*temperature, salmon*forest	4367.54	0	0.60
	2	salmon, temperature, salmon*temperature	4368.34	0.80	0.40
	3	salmon, temperature, precipitation, salmon*temperature, salmon*precipitation	4379.01	11.47	0.00
	4	salmon, temperature, salmon*temperature, salmon*upstream	4381.63	14.09	0.00
	5	null (intercept only)	4390.26	22.73	0.00
	6	temperature, precipitation, temperature*precipitation	4390.94	23.41	0.00
	7	salmon, salmon*forest	4391.44	23.90	0.00
	8	salmon	4392.14	24.61	0.00
	9	temperature	4394.52	26.98	0.00
	10	salmon, salmon*upstream	4394.98	27.44	0.00
δ^15^N					
	1	forest, upstream, forest*upstream	281.20	0.00	0.77
	2	growth index, forest, upstream, forest*upstream	284.53	3.23	0.15
	3	forest, upstream, salmon, forest*upstream	286.89	5.68	0.05
	4	growth index, forest, upstream	287.40	6.20	0.03
	5	forest	294.03	12.83	0.00
	6	growth index, forest, upstream, forest*upstream, salmon*growth index	296.70	15.49	0.00
	7	forest, upstream	297.28	16.09	0.00
	8	growth index, forest	297.28	16.09	0.00
	9	upstream	298.82	17.61	0.00
	10	salmon, forest	299.66	18.46	0.00

Model predictors are annual salmon abundance (salmon), mean maximum temperature (ºC) for the biological year (temperature), distance from stream (forest), distance upstream (upstream), total precipitation for the biological year (precipitation) and derived annual tree ring width (growth index; see methods). Models were ranked using AICc values. For predictors of the growth index, the top two models were averaged. * interaction effect.

**Table 2:** **Sum parameter weights (ω**_**i**_**) for the confidence set of candidate models for growth index (top two models based on model averaging) and δ**^**15**^**N isotopic enrichment models (top three models; ω**_**i **_**=95**%**), ranked using AICc**

**Response variable**	**Parameter**	**Sum ω**_**i**_
Growth index		
	salmon	0.998
	temperature	0.998
	salmon*temperature	0.998
	salmon*forest	0.597
δ^15^N		
	forest	0.963
	upstream	0.963
	forest*upstream	0.963
	growth index	0.146
	salmon	0.045

* Interaction effect.

**Table 3:** Parameter estimates and associated statistics for the best-supported growth index model, as done by model averaging for the top two models

**Response variable**	**Parameter**	**Estimate**	**Std. error**	**95% CI upper lower**	
Growth index					
	intercept	−0.005	0.00	−0.01	0.00
	salmon	0.081	0.01	0.10	0.06
	temperature	0.001	0.00	0.00	0.00
	salmon*temperature	0.086	0.01	0.10	0.07
	salmon*forest	0.074	0.00	0.08	0.07

*Interaction effect.

### Predictors of δ^15^N using GLMMs

In contrast to the predictors of growth, the two spatial variables and their interaction best predicted δ^15^N isotopic enrichment in individual tree rings (1948-1989; Table 1). Summing parameter weights across the top three best-supported models (>95% ω_i_) demonstrated the high importance of the spatial predictors of distance into the forest, distance upstream and their interaction with far lower support for the salmon and growth (Table 2). Distance into the forest, distance upstream and their interaction negatively all had negative parameter estimates (Table 4). The interaction between distance upstream and into the forest is interpreted as having a lesser importance of distance into the forest with distance upstream (Table 4). The two spatial predictors (distance into the forest and distance upstream) were significant (p < 0.05; Table 4). Although four of the 14 trees yielded a significant positive covariation between isotopic signatures and salmon abundance, this effect is accentuated on trees occurring at low carcass densities. Notably, our model-based inference did not identify salmon abundance as an important predictor. We also ran the GLMM using the full temporal complement (1948-2000) in order to include the last decade of high nitrogen levels in the sapwood and found the identical top-ranked models relative to the original model-based selection (Table 1).

**Table 4:** **Parameter estimates and associated statistics for the best-supported δ**^**15**^**N isotopic enrichment model**

**Response variable**	**Parameter**	**Estimate**	**Std. error**	**F**	**p value**
δ^15^N					
	intercept	0.32	0.14	3.63	0.070
	forest	−0.68	0.15	9.03	0.006
	upstream	−1.76	0.59	8.96	0.007
	forest*upstream	−0.97	0.77	1.57	0.223

* Interaction effect.

## Discussion and conclusions

The spatial patterns of carcass distribution, with declining carcasses with distances upstream and into the forest, delineated our study area and provided the spatial framework for our analysis. Of the multiple predators and scavengers observed at Bag Harbour, Haida Gwaii, during the five separate salmon spawning runs (1992, 1993, 1994. 1998, 2000), black bears were the dominant species responsible for the movement of salmon from the stream into riparian zones [5]. In 1993, eight bears uploaded 3100 salmon (10,700 kg) to the riparian zone [32] and consumed approximately one-half of each carcass leaving 330 g of carcass remnants/m^2^ or 10 g N/m^2^[6]. These estimates are similar to those documented in Alaska, where Gende et al. [33] estimated that bears contributed 5 mg N/m^2^ to 10 m strip adjacent to a salmon stream. In addition, brown bears contributed approximately 5 mg N/m^2^ in a 500 m region adjacent to an Alaskan stream, of which 97% was added via urine [15]. In addition to nitrogen provided by salmon carcasses, this input was estimated to account for 10-25% of the forest nitrogen budget [15]. Despite the quantitative estimates for quantity and spatial extent of carcasses, we note that abundance and spatial extent of the carcass distribution at Bag Harbour probably comprises only a fraction of historical levels; in the late 1940s and early 1960′s, the size of the spawning run was approximately seven times the abundance in 2000. Further, the bear trails that occurred beyond the high carcass zone and above the upper reaches of the spawning area [6] broaden the potential contribution of urine-derived nitrogen at much greater distances than examined.

Adjacent to the main spawning area, where salmon carcass densities were highest, we also found high annual radial tree growth. However, high growth was also found further into the riparian zones where carcasses were much less abundant but bear trails and assumed urine deposition were prevalent. Other studies on the annual growth of Alaska Sitka spruce (based on basal area) that grew adjacent to salmon streams found that these trees grew at more than three times the rate of trees at reference sites above waterfalls of unknown distance from the salmon spawning areas [17]. This elevated productivity of trees led to further examinations of the temporal relationship between salmon abundance and tree growth at the watershed level. After an initial investigation [28], an examination of nine sites detected positive relationships between tree ring growth and salmon abundance at five locations, which allowed for the historical reconstruction of salmon runs at five locations [18].

Similar to Drake and Naiman [18], our results generated using indexed growth clearly suggest that salmon abundance has a positive influence on tree growth. However, instead of using reference sites located at a distance (2-25 km;18) , we used within-watershed trees across a sharp ecological gradient from high to very low or zero salmon-nutrient loading. Relying on model-based inference, we found that the abundance of salmon and temperature are major predictors of the tree growth index, with all other predictors consisting of salmon abundance and its interaction with temperature and distance into the forest. Interestingly, the interaction between salmon and distance into the forest was positive. Initially considered counterintuitive, this positive relationship may be the result of elevated nitrogen levels in areas with higher salmon loading where, as a consequence, trees may not always be nitrogen-limited. Their growth, although high, may not correlate as closely with salmon as trees that are more nitrogen-limited and experience lower levels of salmon-derived nitrogen. Considered initially as controls, the growth response of trees at larger distances from the spawning gravel beds (less than 800 m) to salmon abundance provides evidence that the spatial effects of salmon on tree growth extend at least 90 m into the riparian zone and likely further. Temperature and the interaction between temperature and salmon abundance were also highly ranked as predictors, suggesting that differencing techniques for the removal of environmental signals from a chronology (e.g. [18]) may, at the minimum, remove some of the influences of salmon and subsequently alter reconstructions.

Patterns of δ^15^N enrichment tracked what is known of the spatial transfer of salmon into the forest, with higher levels of enrichment found adjacent to the spawning area and declining with distance into the forest and above the upper reaches of the spawning zone. Not surprisingly, the use of GLMM confirmed our visual assessment; spatial variables were found to be the strongest predictors of δ^15^N at Bag Harbour. This finding is similar, although on a much finer spatial resolution, to Hocking and Reimchen [34], who found that chum salmon abundance was a leading predictor of δ^15^N variation in plants and invertebrates at the watershed level. Similarly, Hilderbrand et al. [15] found that not only δ^15^N signatures of white spruce decline with distance from salmon-spawning streams, but that the activity of radio-collared brown bears were highly correlated with this same gradient of depletion.

The mechanism of the influence of salmon on tree growth is generally considered to be related to the input of salmon-derived nitrogen to nitrogen-limited trees. In addition to growth being positively influenced by salmon abundance, δ^15^N derived from salmon and subsequently laid down in annual tree rings may provide an additional proxy for salmon abundance. An experimental study that monitored the uptake of δ^15^N by western redcedar trees (*Thuja plicata*) found that δ^15^N uptake in the fall was distributed to leaves and stems in the spring, which substantiates our use of a one year lag effect [24] but also furthers the idea that salmon-derived δ^15^N could be linked to salmon abundance, similar to growth. Studies of natural δ^15^N levels in tree rings have suggested that they may reflect patterns of atmospheric nitrogen deposition (e.g. [35]) and fertilization studies have demonstrated that tree ring δ^15^N changes may reflect the historical N regime in a forest [36]. Kiernan and Johnson [21] found relatively weak support for the idea that δ^15^N levels in tree rings can be linked to salmon abundance and suggested that nitrogen dynamics in the adjacent stream, soil and within-tree may have acted to obscure their ability to discriminate salmon-derived and autochthonous sources of N.

Major factors that remain poorly understood, and which potentially confound the use of δ^15^N levels as a predictor of salmon abundance in this study and others, include denitrification, nitrogen isotope fractionation, translocation of N isotopes across tree rings (e.g. [35,36]) and local soil and stream nitrogen dynamics [21,37]. In particular, denitrification, where ^14^ N is preferentially lost in the system and higher proportions of ^15^ N remain, could be particularly problematic if it varied with spatial patterns of salmon loading (e.g. [38]). Similar δ^15^N declines with distance away from the salmon spawning area (up to 150 m) have been previously attributed to salmon-derived nutrients but these conclusions were strengthened by a lack of similar declines on nearby non-salmon bearing streams [39]. While we have no prior expectation that denitrification varied spatially over the study site we cannot discriminate denitrification from salmon loading using δ^15^N if the two are similar.

The close spatial coupling between the distribution of salmon carcasses at Bag Harbour and the δ^15^N signatures of trees, particularly the steep gradients in signatures (5-10%) over short distances (50 m), suggest that tree ring δ^15^N signatures in this watershed may be proxies for historical nutrient uploading in this watershed. Although there are a number of feasible alternative explanations, we make several possible inferences if these δ^15^N patterns are linked to salmon. In the riparian zone adjacent to the current major spawning and bear feeding areas, there has been general temporal continuity of the nutrient uploading during the last five decades. Yet, in the upper reaches of the watershed, between the present spawning zone and the headwater lake, and in two trees further downstream in the low carcass zone, where there is no current salmon transfer, δ^15^N signatures were historically equivalent to those observed in the major feeding areas, but have become depleted by approximately 5‰ by 2000, suggesting a historically wider distribution of salmon-derived nutrients. Consistent with this suggestion, there was also a depletion in yearly isotopic signatures, relative to historic periods, and which are more similar to the yearly variability in trees in present high carcass densities. There was a limited isotopic signature of the two major pulses of salmon recorded in the late 1940s and early 1960′s apart from two of the low-carcass density trees (BH5 and BH30). Bears abandon a greater number of carcasses and consume a smaller proportion of each carcass when salmon escapement is relatively high [6] yet it is likely that numbers of bears in a watershed are determined on average annual salmon escapement, rather than yearly fluctuations. If so, this would decouple any immediate covariation and reduce the chances of detecting year to year fluctuations, thus limiting the application of isotopic proxies for salmon abundance to a longer time frame.

Tree ring information has been used as a proxy for constructing historical salmon abundances at multiple watersheds with riparian zone stand chronologies and nearby salmon-free controls [18] and at the regional level in climate-sensitive series [40], both with some success. Similar techniques have been employed to examine historical seabird activity extending from the input of guano to forested nesting colonies [41,42]. Our study, although not completing salmon reconstructions and offering only a relatively short time-scale analysis, provides insight into the use of growth indexes and tree ring δ^15^N as proxies for historical salmon abundance. We observed that δ^15^N signatures exhibit a relatively consistent decrease from large to small trees both in high and low carcass density zones allowing calibration of δ^15^N in cores that extend from the cambium towards the center. Without a dbh-correction, δ^15^N values tend to remain somewhat stable over time. Yet, because we detected a strongly positive relationship between tree dbh and δ^15^N and subsequently used this relationship as a calibration factor, δ^15^N values demonstrate a decline over time. We also found elevated isotopic signatures in the late 1940′s and 1950′s of several trees that are currently in low carcass zones, suggesting elevated salmon uploading at that time. This size factor has consequences for potential use and improvement of salmon abundance reconstructions (e.g. [21]), although further investigation is required.

Our study, which reaffirms the importance of salmon to riparian productivity and contributes new insight for the complex, spatiotemporal influences of salmon abundance, temperature and tree location relative to salmon upon conifer growth and δ^15^N levels. Both of these variables provide strong spatial indicators. The use of δ^15^N for temporal reconstructive purposes remains challenging. We speculate that the appropriate choice of trees in riparian zones across ecological gradients of salmon carcass densities and bear activity and the isotopic correction for yearly changes in tree size may allow improved assessment of tree ring growth as a historical proxy for salmon abundance and distribution during previous centuries in watersheds where ancient trees still persist.

## Abbreviations

AICc: Aikaike’s information criteria for finite sample sizes; BH: Bag harbour; dbh: Diameter at breast height; GLMM: General linear mixed model.

## Competing interests

The authors declare that they have no competing interests.

## Authors’ contributions

TR conceived of, carried out fieldwork and statistical analyses for the study. CF completed tree ring measurements, data collection and GLMM statistical analyses. Both authors participated in drafting the manuscript and have read and approved the final manuscript.
